# Evolution of Frozen Section in Carcinoma Breast: Systematic Review

**DOI:** 10.1155/2022/4958580

**Published:** 2022-05-23

**Authors:** Manjit Kaur Rana, Amrit Pal Singh Rana, Uttam Sharma, Tushar Singh Barwal, Aklank Jain

**Affiliations:** ^1^Pathology/Lab Medicine, All India Institute of Medical Sciences, Bathinda, 151001 Punjab, India; ^2^General Surgery, All India Institute of Medical Sciences, Bathinda, 151001 Punjab, India; ^3^Department of Zoology, Central University of Punjab, Bathinda, 151401 Punjab, India

## Abstract

**Background:**

The frozen section (FS) has been a good technique in surgical management of breast lesions since many years. But complete agreement and cooperation have not been achieved everywhere among surgeons and pathologists especially in the developing countries. FS undergoes continuous criticism due to various shortcomings but continued to be evaluated especially in developing countries.

**Objectives:**

This review was conducted to synthesize information on the use of frozen section in carcinoma breast. *Data Sources*. The MEDLINE database for frozen section since its origin and its implication in recent breast surgery techniques was studied. *Study Eligibility Criteria*. Sixty-five articles were reviewed with complete analysis on FS in both benign and malignant breast lesions. *Study Appraisal and Synthesis Methods*. The analysis of frozen section was done as a diagnostic tool in breast lesions, margin status in breast conservative surgery in carcinoma breast, and sentinel lymph node and use of immunohistochemistry for sentinel lymph node FS.

**Results:**

It was analysed that the FS gives accurate results in margin status analysis, decreasing rerecurrence.

**Conclusion:**

The accuracy of FSA, low recurrence rate, avoidance of reoperation, and good cosmesis are the key points of its use in breast conservative surgery. Its use in sentinel lymph node biopsy (SLNB) is equivocal. However, application of immunohistochemistry on frozen section of SLNB is an evolving trend in today's era.

## 1. Introduction

FS is an important part of breast surgery with its earliest application since 1891. Various review reports have been published on accuracy rates of FS ranging from 94% to 99% [1–4]. FS is required for assessment of margin status and sentinel lymph node status, and in addition to this, fresh frozen tissue is also required for supplementary testing. The need for axillary lymph node dissection (ALND) in patients with small metastases has been recently called into question. Sentinel lymph node biopsies (SLNBs) evaluated intraoperatively by frozen section may influence the need for further axillary dissection (AD) [5, 6]. Literature was reviewed, and data was collected to analyse the role of frozen section in breast cancer surgery. Studies showing variations and trends were analysed for the accuracy and use of FS in the diagnosis of carcinoma breast and its need for use in assessing the margin and sentinel lymph node status in breast conservative surgery.

## 2. Frozen Section as a Diagnostic Tool in Breast Lesions

FS analysis being an essential part of breast surgery had been utilised by Welch in 1891 for the diagnosis of benign tumors of the breast [1]. Initially, the processing of frozen tissue was the most common limitation among the major shortcomings of the method and its outcomes. The detailed technique of FS was mentioned by Cullen and was adapted as a diagnostic tool by Wilson at the Mayo Clinic in 1905 [7, 8]. Frozen section has been criticized repeatedly since its origin for false-positive and false-negative (FN) results of the diagnosis and for being a difficult and demanding technique. The diagnosis of malignancy mistakenly made on borderline tumors leads to an unnecessary radical operation; therefore, the FS method was not considered a preferred method over biopsy [9–11]. Further studies done also showed that the careful processing of FS resulted in the same diagnostic qualities of slides made by the frozen and paraffin methods with few exceptions. But surgical revision of cases of frozen section diagnoses after a final study of paraffin sections always resulted in conflicts between the surgeon and the pathologist for under- or overdiagnosis of carcinoma breast, delayed reporting by pathologist, and inadequate sampling by surgeon [12].

However, a study conducted by Rosen emphasised that FS analysis is accurate in the diagnosis of infiltrating ductal cell carcinoma [13]. In the early 80s, frozen sections of core needle/open biopsy specimens and cytologic smears of breast mass aspirates were considered acceptable methods for the rapid and accurate initial evaluation. In an equivocal opinion given by Bauermeister, it was concluded that as negative report is always tenuous and should not lead to definitive therapy so selection of the patient for any type of procedure should be done depending upon the clinical situation [14]. Sixteen studies reported in the literature dealing with the accuracy of the frozen section method for the diagnosis of breast lesions were reviewed. Data collected was compiled and compared. The composite of these studies comprises 11,632 FS examinations of breast tissue (Table 1). There were 1.85% deferred (DFD) diagnoses, 1.06% FN diagnoses, and 0.36% false-positive (FP) fielding an overall accuracy of 97.28% for the method.

Many studies found FS as a highly accurate method for the breast lump diagnosis. It was suggested that this may be applied to open biopsy or core needle core biopsy (CNB) specimens and in either case the false-negative rate was found less than that experienced with needle aspiration. An analysis done by Santos et al. showed that frozen section and histopathological findings had excellent correlation in case of analysis of the fragments of palpable breast tumors obtained by CNB with 98.2% accuracy [32]. So, it was favoured that FS results of CNB in case of palpable tumors and suspected breast cancer have good histopathological concordance. But limited data is available to evaluate the accuracy of FS analysis and ultrasound-guided CNB of the nonpalpable breast lesions. Few studies done have shown good sensitivity/specificity characteristics and 98.3% accuracy [26]. Data reviewed have shown high accuracy rates of FS as diagnostic tool except in some cases, and FS is a prominent point of intersection between surgeons and pathologists. But it should not be used as a shortcut to a definitive diagnosis [33]. In our experience, only 7/55 (12.7%) fresh tissues were received for primary diagnosis of the breast lesion with 94.4% specificity and 100% sensitivity.

## 3. Frozen Section and Margin Status in Breast Conservative Surgery in Carcinoma Breast

Though assessment of margins using permanent section evaluation is the standard method of ensuring complete tumor excision in carcinoma breast surgery (BCS), FS is a good tool for decision-making at the time of surgery. If the margin is positive, surgical reexcision can be done to reduce the likelihood of subsequent local recurrence. The use of FS in margin status in case of (BCS) showed 6.3%-26.9% reduction in two-stage surgery (Table 2) [14, 27, 34–38].

In our practice also, in 27/55 (49%), FS was performed for margin status and showed 100% specificity and 100% sensitivity. Intraoperative frozen section analysis helped in managing all the cases in a single-stage surgery. Bauermeister (1980) in his work experienced a modification in technique with intraoperative circumferential FS analysis, and no significant changes in results were found. However, only 5.0% FN rate was noticed because multiple tumors, invasive lobular carcinoma, large tumor size, and multiple excisions increased the chances of conversion of BCS to mastectomy [14]. Chagpar et al. observed that, in patients with DCIS, gross pathological examination and radiographically sliced specimen significantly affect intraoperative margin assessment [39]. However, Riedl and fellows retrospectively analysed that 9% of all the cases within situ and pT1 lesions had to undergo a two-stage operation due to FN intraoperative FS results and only 1.2% local recurrence rate with one-year follow-up. Where there is no intraoperative assessment of margin status, rates of reoperation in general have been reported to be in excess of 18.0% in case of IDC and 29.5% in case of DCIS. The need for reoperation after FN FS analysis was due to DCIS, size of the tumor predominantly [40]. One study of Jorns et al. including 25 patients has mentioned good results of FS analysis in the margin status of DCIS [41]. With increasing interest in reducing the reoperation rates in BCS, other intraoperative modalities also come in competition with FS such as imprint cytology (IC) and imaging techniques. However, Osborn et al. have suggested that FS is cost-effective only when there are reexcision rates of more than 36% in an institution [42]. A systematic review including IC and FS done by Esbona and fellows showed decreased reoperation rates from 26 to 4% for IC and from 27 to 6% for FS [43]. Another systematic review done by Butler-Henderson and associates showed that FS and IC added an average 20-30 minutes to operation times whereas an ultrasound probe delivers results in a timely manner but has a limited role in cases with DCIS and multifocal cancer [44].

In spite of the impact of other modalities in intraoperative assessment of margins, FS continued to flourish. Emmadi and Wiley observed that negative margins especially <2 mm thickness carried a >25% risk of residual disease and recommended agreement with breast cancer summary protocols of the College of American Pathologists (CAP) in documenting the measurement of clearance at the closest margins in addition to only positive/negative margin status [45]. Furthermore, margin assessment was improved with multiple side sampling. Tan and fellows performed BCS with six margins of the excised breast tissue, and only 2.4% reoperation was observed in 4 out of 161 cases including one FN case and three of missed multicentric disease [46]. But margin analysis has technical difficulty of freezing the tissue and hence resulted in high FN rates; nonetheless, it still manages to reduce the reexcision rate if multiple tissues are sampled. The limitations of routine FSA for margin status include time resource allocations, labour intensity, technical challenges, and cost considerations [41, 47]. All over, intraoperative FS margin analysis helps in reducing number of reoperations in patients undergoing BCS. This method has significant implications for patient satisfaction and cost of care [48]. Systematic review and meta-analysis data suggested that frozen section and cytology have the greatest diagnostic accuracy [49]. However, these methods are resource-intensive and turnaround times for results have prevented widespread international adoption. Emerging technologies need to compete with the diagnostic accuracy of existing techniques while offering advantages in terms of speed, cost, and reliability.

## 4. Frozen Section, BCS, and Sentinel Lymph Node

Axillary lymph node involvement is considered the most important prognostic factor in early-stage BC. A total of 22/55 FSs for axillary lymph node status were reported with 100% specificity and 100% sensitivity for macrometastasis with treatment for early-stage BC further evolving with evolution of sentinel lymph node biopsy (SLNB) along with breast imaging, radiation, and other treatments. Integration of SLNB as a standard point of care for the patient was the important and first step towards sparing the complications of ALND with a clinically negative axilla [50, 51]. Consequently, diagnosis of SLNBs with frozen section became common practice, with up to 35% of sentinel lymph nodes (SLNs) with metastasis requiring further ALND [49]. Retrospective intraoperative FS analysis of the SLN done by Francissen et al. on 628 patients showed a high FN rate of 16.1%. Only 12.4% of the patients benefited from intraoperative FS, as secondary ALND could be avoided. It was observed that FS may be indicated for a selected group of patients [52].

Hashmi et al. performed a retrospective study on 154 patients. The SLNs were sectioned at 2 mm intervals and submitted entirely for frozen sections followed by histopathological examination of three levels of each section submitted. The sensitivity and specificity of frozen section analysis of SLN for the detection of macrometastasis were found to be 100% while those for micrometastasis were 33.3% and 100%, respectively [53].

In a review done by Poling et al., 1940 cases of FS were assessed for SLNB. FN results were seen in 95 cases (4.9% of total cases, 23.8% of positive node cases) with majority of missed metastases including isolated tumor cells or micrometastases. SLNBs evaluated intraoperatively by frozen section may impact the need for further AD; as in the later years, a trend of completion of AD was faced after a discrepant frozen SLNB. SLNBs may be unnecessary, and furthermore, it can compromise tissue for further study [54].

Despite an online appendix (2014) by ASCO addressing the known limitations of frozen section diagnosis, guidelines did not encourage or discourage the use of frozen section diagnosis for SLNB [55].

Retrospective monocentric study done by Hoen et al. of 293 patients operated on stage pT1 or pT2 breast cancer with SLNB showing a false-negative rate of 13.5%. Intraoperative frozen sections benefited only 12.8% of the patients who had their full lymph node dissection at the same surgery. The intraoperative frozen section of SLNB benefits a limited number of patients, and preoperative axillary ultrasound examination was suggested [56].

The trial of the American College of Surgeons Oncology Group Z0011 showed that early-stage breast cancer with limited sentinel node metastasis patients treated with breast conservation and systemic therapy did not show advantage from axillary lymph node dissection, hence resulting in a decline in the use of frozen section in the diagnosis of SLNs. Jorns et al. identified 116 pre-Z0011 and 134 post-Z0011 patients. There was post-Z0011 decline in ALND (*P* = 0.014), and SLN positivity was associated with larger (≥1.6 cm) tumor size (*P* = 0.002). These findings supported reduced requirement for SLN FS for BCS patients post-Z0011 [57]. Bishop and associates conducted a study to determine the effect of the Z0011 trial on utility of intraoperative sentinel lymph node evaluation and observed that, during the pre-Z0011 years, FS of SLNB was done in 22/22 cases (100%) in 2009 and 15/22 cases (68%) in 2010. In the post-Z0011 years from 2011 to 2015, FS of SLNB was sent only in 3/151 cases (2%) and it was observed that 28/151 (19%) revealed metastatic deposits in SLNB on histopathological examination. Therefore, routine frozen section diagnosis for SLNB biopsies can be avoided in these patients [58].

Lombardi and fellows perceived a low and good sensitivity of FS in detecting micrometastases (19%) and macrometastases (75%), respectively, with mainly FN in smaller metastases (mean 2.1 mm), more probable in infiltrating lobular carcinoma cases [59].

In a study done by Russo and fellows, a total of 281 patients were evaluated. Macrometastasis (13.1%) and micrometastasis (66.7%) (*P* < 0.001) were found in FN cases [60].

Z0011 criteria applied as a standard in management of axilla lead to a significant decline in intraoperative FS diagnosis of SLNBs of patients with cT1 to T2 cN0 stage and resulted in a substantial decrease in ALND in a large proportion of patients [58].

It was observed that eliminating routine FS diagnosis for SLNB in BCS patients is acceptable and cost-effective, especially when considering prolonged anaesthesia time and associated waiting time for FS results and leading to optimal use of resources in pathology departments.

## 5. Use of Immunohistochemistry for Sentinel Lymph Node FS

At our institution, rapid IHC was performed on random FSs of breast tissue for research purpose and total turnaround time observed was 15-20 minutes with 100% sensitivity and specificity. As mentioned in the literature, Chao (2004) supported intraoperative pathologic evaluation of the SLN as option of complete ALND in two-thirds of carcinoma breast cases with nodal disease. It was suggested that false-positive FS may result in unnecessary morbid ALND completion and one must be aware of the complications. However, in one-third of carcinoma breast cases with FN results, cytokeratin (CK) staining on paraffin-embedded tissue section improved the sensitivity but there was no clinically significant effect of immunohistochemically positive cells on therapy, hence warranting the use of immunohistochemistry (IHC) and necessitating the prospective randomized trials to show its clinical significance [61].

Salem and associates in year 2006 tried intraoperative IHC staining of touch imprints of axillary sentinel nodes and found feasible and a reliable method for evaluating axillary nodes [62]. Choi et al. (2006) and Krishnamurthy et al. (2009) experimented ultrarapid IHC with mean turnaround time of 20 min on 178 SLNBs. The sensitivity rates of frozen H&E staining (70.0%) and ultrarapid IHC (85.0%) were found to be statistically insignificant. Although 100% specificity was observed for each method, ultrarapid IHC benefitted one case of micrometastasis and two cases of isolated tumor cells (ITCs) only. So ultrarapid cytokeratin immunohistochemistry (CK-IHC) upgraded the rapid intraoperative detection of sentinel node micrometastasis and ITCs in breast cancer [63, 64].

**Figure 1:**
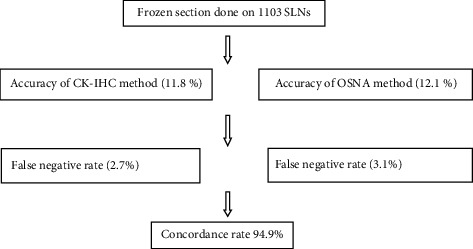
Flowchart showing comparison of two methods used to detect metastases in SLNs [67].

Stovgaard et al. also studied that IHC on frozen section leads to the detection of more ITC and MIC intraoperatively [65]. Furthermore, Cserni (2012) reviewed the use of one-step nucleic acid amplification (OSNA) automated molecular assay based on the quantification of cytokeratin 19 mRNA and found 96% concordance rate with histopathology and IHC. But then, Shigematsu and fellows compared cytokeratin immunohistochemistry (CK-IHC) assay on FSs and OSNA assay of the whole node in intraoperative evaluation for SLN metastases in patients with invasive breast cancer. Hence, this suggested that both assays had compatible diagnostic capacities and can be used as reliable techniques for intraoperative diagnoses of SLN metastases in breast cancer patients (Figure 1) [66, 67].

## Figures and Tables

**Table 1:** Comparison of frozen section results in the diagnosis of breast lesions.

Sr. no.	Author and year	No. of cases	DF cases	FN	FP	Accuracy
1	Jenning et al., 1957 [15]	212	5.7%	0.9%	Nil	93.4%
2	Akermen et al., 1958 [16]	440	2.2%	0.9%	Nil	96.8%
3	Winship et al., 1959 [17]	1004	1.17%	0.8%	Nil	98.1%
4	Rosen, 1978 [13]	556	5.3%	1.4%	Nil	93.2%
5	Kagali, 1983 [18]	158	1.2%	1.2%	1.8%	94.9%
6	Fessia et al., 1984 [19]	4436	Nil	1.6%	Nil	96.5%
7	Rogers et al., 1987 [20]	315	1.2%	2.8%	Nil	95.9%
9	Hou et al., 1995 [21]	549	Nil	0.5%	Nil	99.1%
10	Altaf, 2004 [22]	203	4.9%	1.9%	Nil	98%
11	Sultana et al., 2005 [23]	316	2.1%	0.3%	0.6%	99%
12	Karve et al., 2005 [24]	237	0.8%	0.4%	Nil	99.5%
13	Mulleller Holzner et al., 2007 [25]	2619	Nil	0.4%	0.08%	99.5%
14	Brunner et al., 2009 [26]	120	Nil	3.3%	Nil	96.7%
15	Belliolo et al., 2009 [27]	290	Nil	0.59%	Nil	99.3%
16.	Mahadevappa et al., 2017 [28]	62	1.6%	Nil	1.62%	98.3%
17.	Kaira et al., 2018 [29]	115	3.5%	Nil	1.7%	98.3%
18.	Grabenstetter et al., 2019 [30]	711	1.1%	5.4%	Nil	96.0
19.	Namdar et al., 2021 [31]	1742	Nil	1.1%	5.1%	93.6%
Total		14,085	2.41%	2.52%	1.82%	95.62%

**Table 2:** Comparison of frozen section results in the margin status of breast carcinoma.

Sr. no.	Author, year	Margins	Positive	Reexcision	Mastectomy
1	Bauermeister, 1980 [14]	446	14.3%	6.3%	5.1%
2	Sauter et al., 1994 [34]	359	9.4%	6.6%	2.8%
3	Weber et al., 1997 [35]	140	15%	8.7%	6.3%
4	Aziz et al., 2006 [36]	1430	14.3%	10.6%	3.7%
5	Belliolo et al., 2009 [27]	258	18%	18%	Nil
6	Dener et al., 2009 [37]	190	16%	12.7%	3.3%
7	Osako et al., 2015 [38]	1029	30.3%	26.9%	1.4%

## Data Availability

The data used to support the findings of this study are included within the article.

## References

[1] Wright J. R. (1985). The development of the frozen section technique, the evolution of surgical biopsy, and the origins of surgical pathology. *Bulletin of the History of Medicine*.

[2] Jeevan R., Cromwell D. A., Trivella M. (2012). Reoperation rates after breast conserving surgery for breast cancer among women in England: retrospective study of hospital episode statistics. *BMJ*.

[3] Unzeitig A., Kobbermann A., Xie X. J. (2012). Influence of surgical technique on mastectomy and reexcision rates in breast- conserving therapy for cancer. *International Journal of Surgical Oncology*.

[4] Tamanuki T., Namura M., Aoyagi T., Shimizu S., Suwa T., Matsuzaki H. (2020). Effect of intraoperative imprint cytology followed by frozen section on margin assessment in breast-conserving surgery. *Annals of Surgical Oncology*.

[5] Almarzooq R., Alrayes A., Saeed A., Abdulla H. (2018). Accuracy of intraoperative frozen section evaluation of sentinel lymph node biopsy in breast cancer: our experience in Bahrain. *The Gulf Journal of Oncology*.

[6] Giuliano A. E., Ballman K. V., McCall L. (2017). Effect of axillary dissection vs no axillary dissection on 10-year overall survival among women with invasive breast cancer and sentinel node metastasis: the ACOSOG Z0011 (Alliance) randomized clinical trial. *Journal of the American Medical Association*.

[7] Cullen T. S. (1895). A rapid method of making permanent sections from frozen sections by the use of formalin. *Johns Hopkins Hospital Bulletin*.

[8] Wilson L. B. (1905). A method for the rapid preparation of fresh tissues for the microscope. *Journal of the American Medical Association*.

[9] Juan R. (1996). Ackerman’s surgical pathology. *Introduction: Frozen Section*.

[10] Waldemar A. (1983). Principles and techniques of surgical pathology. *Schmidt. The Intraoperative Consultation*.

[11] Godazandeh G., Alizadeh-Navaei R., Shamshirian A., Heydari K., Shojaee L. (2021). Diagnostic value of intraoperative frozen section in breast-conserving surgery: a systematic review and meta-analysis. *International Journal of Cancer Management*.

[12] Teloh H. A. (1957). *Methods in surgical pathology*.

[13] Rosen P. (1978). Frozen section diagnosis of breast lesions. Recent experience with 556 consecutive biopsies. *Annals of Surgery*.

[14] Bauermeister D. E. (1980). The role and limitations of frozen section and needle aspiration biopsy in breast cancer diagnosis. *Cancer*.

[15] Jennings E. R., Landers J. W. (1957). The use of frozen section in cancer diagnosis. *Surgery, Gynecology & Obstetrics*.

[16] Ackerman L. V., Ramirez G. A. (2005). The indications for and limitations of frozen section diagnosis; a review of 1269 consecutive frozen section diagnoses. *The British Journal of Surgery*.

[17] Winship T., Rosvoll R. V. (1959). Frozen sections. An evaluation of 1810 cases. *Surgery*.

[18] Kagali V. A. (1983). The role and limitations of frozen section diagnosis of palpable mass in the breast. *Surgery, Gynecology & Obstetrics*.

[19] Fessia L., Ghiringhello B., Arisia R., Bolta G., Aimone V. (1984). Accuracy of frozen section diagnosis in breast cancer detection: a review of 4436 biopsies and comparison with cytodiagnosis. *Pathology, Research and Practice*.

[20] Rogers C., Klatt E. C., Chandrasoma P. (1987). Accuracy of frozen-section diagnosis in a teaching hospital. *Archives of Pathology & Laboratory Medicine*.

[21] Hou M. F., Huang T. J., Lin H. J. (1995). Frozen section of diagnosis of breast lesions. *Gaoxiong Yi Xue Ke Xue Za Zhi*.

[22] Altaf F. J. (2004). Audit of breast frozen sections. *Annals of Saudi Medicine*.

[23] Sultana N., Kayani N. (2005). Validity of frozen section in the diagnosis of breast lumps: 5 years experience at the Aga Khan University Hospital. *The Journal of the Pakistan Medical Association*.

[24] Karve P. V., Jambhekar N. A., Desai S. S., Chinoy R. F. (2005). Role of frozen section evaluation in patient with breast lumps: a study of 251 cases. *The Indian Journal of Surgery*.

[25] Mueller-Holzner E., Frede T., Daniaux M. (2007). Ultrasound-guided core needle biopsy of the breast: does frozen section give an accurate diagnosis?. *Breast Cancer Research and Treatment*.

[26] Brunner A. H., Sagmeister T. H., Kremer J., Riss P., Brustmann H. (2009). The accuracy of frozen section analysis in ultrasound- guided core needle biopsy of breast lesions. *BMC Cancer*.

[27] Enrique Bellolio J., Pablo Guzmán G., Juan Orellana C. (2009). Validez diagnóstica de la biopsia intraoperatoria en cirugía de lesiones mamarias palpables. *Revista Médica de Chile*.

[28] Mahadevappa A., Nisha T. G., Manjunath G. V. (2017). Intra-operative diagnosis of breast lesions by imprint cytology and frozen section with histopathological correlation. *Journal of Clinical and Diagnostic Research*.

[29] Kaira V., Gupta A. K., Agarwal A., Kala S., Kaira P. (2018). Frozen section versus paraffin section in diagnosis of breast lesions: a comparative study. *Clinical Cancer Investigation Journal*.

[30] Grabenstetter A., Moo T. A., Hajiyeva S. (2019). Accuracy of intraoperative frozen section of sentinel lymph nodes after neoadjuvant chemotherapy for breast carcinoma. *The American Journal of Surgical Pathology*.

[31] Namdar Z. M., Omidifar N., Arasteh P. (2021). How accurate is frozen section pathology compared to permanent pathology in detecting involved margins and lymph nodes in breast cancer?. *World Journal of Surgical Oncology*.

[32] Santos R. L. C. D., Lasmar R. B., Fontes T. M. P., Fonseca R. D. C. S. D. P., Saldanha P. D. A. B., Santos R. F. C. D. (2014). Percutaneous core biopsy of palpable breast lesions: accuracy of frozen section histopathological exam in the diagnosis of breast cancer. *Revista do Colégio Brasileiro de Cirurgiões*.

[33] Taxy J. B. (2009). Frozen section and the surgical pathologist: a point of view. *Archives of Pathology & Laboratory Medicine*.

[34] Sauter E. R., Hoffman J. P., Ottery F. D. (1994). Is frozen section analysis of reexcision lumpectomy margins worthwhile? Margin analysis in breast reexcisions. *Cancer*.

[35] Weber S., Storm F. K., Stitt J., Mahvi D. M. (1997). The role of frozen section analysis of margins during breast conservation surgery. *The Cancer Journal from Scientific American*.

[36] Aziz D., Rawlinson E., Narod S. A. (2006). The role of reexcision for positive margins in optimizing local disease control after breast-conserving surgery for cancer. *The Breast Journal*.

[37] Dener C., Inan A., Sen M., Demirci S. (2009). Intraoperative frozen section for margin assessment in breast conserving Surgery. *Scandinavian Journal of Surgery*.

[38] Osako T., Nishimura R., Nishiyama Y. (2015). Efficacy of intraoperative entire-circumferential frozen section analysis of lumpectomy margins during breast-conserving surgery for breast cancer. *International Journal of Clinical Oncology*.

[39] Chagpar A., Yen T., Sahin A. (2003). Intraoperative margin assessment reduces reexcision rates in patients with ductal carcinoma in situ treated with breast-conserving surgery. *American Journal of Surgery*.

[40] Riedl O., Fitzal F., Mader N. (2009). Intraoperative frozen section analysis for breast-conserving therapy in 1016 patients with breast cancer. *European Journal of Surgical Oncology*.

[41] Jorns J. M., Visscher D., Sabel M. (2012). Intraoperative frozen section analysis of margins in breast conserving surgery significantly decreases reoperative rates: one-year experience at an ambulatory surgical center. *American Journal of Clinical Pathology*.

[42] Osborn J. B., Keeney G. L., Jakub J. W., Degnim A. C., Boughey J. C. (2011). Cost-effectiveness analysis of routine frozen-section analysis of breast margins compared with reoperation for positive margins. *Annals of Surgical Oncology*.

[43] Esbona K., Li Z., Wilke L. G. (2012). Intraoperative imprint cytology and frozen section pathology for margin assessment in breast conservation surgery: a systematic review. *Annals of Surgical Oncology*.

[44] Butler-Henderson K., Lee A. H., Price R. I., Waring K. (2014). Intraoperative assessment of margins in breast conserving therapy: a systematic review. *The Breast.*.

[45] Emmadi R., Wiley E. L. (2012). Evaluation of resection margins in breast conservation therapy: the pathology perspective—past, present, and future. *International Journal of Surgical Oncology*.

[46] Tan M. P., Sitoh N. Y., Sim A. S. (2014). The value of intraoperative frozen section analysis for margin status in breast conservation surgery in a nontertiary institution. *International Journal of Breast Cancer*.

[47] Jaafar H. (2006). Intra-operative frozen section consultation: concepts, applications and limitations. *The Malaysian Journal of Medical Sciences*.

[48] Boughey J. C., Hieken T. J., Jakub J. W. (2014). Impact of analysis of frozen-section margin on reoperation rates in women undergoing lumpectomy for breast cancer: evaluation of the National Surgical Quality Improvement Program data. *Surgery*.

[49] John E. R. S., Al-Khudairi R., Ashrafian H. (2017). Diagnostic accuracy of intraoperative techniques for margin assessment in breast cancer surgery. *Annals of Surgery*.

[50] Lester S. C., Kumar V., Abbas A. K., Aster J. C. (2010). The breast. *Robbins and Cotran Pathologic Basis of Disease*.

[51] Veronesi U., Paganelli G., Viale G. (2003). A randomized comparison of sentinel-node biopsy with routine axillary dissection in breast cancer. *The New England Journal of Medicine*.

[52] Francissen C. M. T. P., Parra R. F. D. V. L., Mulder A. H., Bosch A. M., Roos W. K. D. (2013). Evaluation of the Benefit of Routine Intraoperative Frozen Section Analysis of Sentinel Lymph Nodes in Breast Cancer. *ISRN Oncology*.

[53] Hashmi A. A., Faridi N., Khurshid A. (2013). Accuracy of frozen section analysis of sentinel lymph nodes for the detection of Asian breast cancer micrometastasis - experience from Pakistan. *Asian Pacific Journal of Cancer Prevention*.

[54] Poling J. S., Tsangaris T. N., Argani P., Cimino-Mathews A. (2014). Frozen section evaluation of breast carcinoma sentinel lymph nodes: a retrospective review of 1, 940 cases. *Breast Cancer Research and Treatment*.

[55] Lyman G. H., Temin S., Edge S. B. (2014). Sentinel lymph node biopsy for patients with early-stage breast cancer: American Society of Clinical Oncology clinical practice guideline update. *Journal of Clinical Oncology*.

[56] Hoen N., Pral L., Golfier F. (2016). Value of intraoperative frozen section of sentinel lymph node in breast cancer. Retrospective study about 293 patients. *Gynécologie, Obstétrique & Fertilité*.

[57] Jorns J. M., Kidwell K. M. (2016). Sentinel lymph node frozen-section utilization declines after publication of American College of Surgeons oncology group Z0011 trial results with no change in subsequent surgery for axillary lymph node dissection. *American Journal of Clinical Pathology*.

[58] Bishop J. A., Sun J., Ajkay N., Sanders M. A. (2016). Decline in frozen section diagnosis for axillary sentinel lymph nodes as a result of the American College of Surgeons oncology group Z0011 trial. *Archives of Pathology & Laboratory Medicine*.

[59] Lombardi A., Nigri G., Maggi S. (2018). Role of frozen section in sentinel lymph node biopsy for breast cancer in the era of the ACOSOG Z0011 and IBCSG 23-10 trials. *The Surgeon*.

[60] Russo L., Betancourt L., Romero G. (2017). Frozen section evaluation of sentinel lymph nodes in breast carcinoma: a retrospective analysis. *E Cancer Medical Science*.

[61] Chao C. (2004). The use of frozen section and immunohistochemistry for sentinel lymph node biopsy in breast cancer. *The American Surgeon*.

[62] Salem A. A., Douglas-Jones A. G., Sweetland H. M., Mansel R. E. (2006). Intraoperative evaluation of axillary sentinel lymph nodes using touch imprint cytology and immunohistochemistry. Part II. Results. *European Journal of Surgical Oncology*.

[63] Choi Y. J., Yun H. R., Yoo K. E. (2006). Intraoperative examination of sentinel lymph nodes by ultrarapid immunohistochemistry in breast cancer. *Japanese Journal of Clinical Oncology*.

[64] Krishnamurthy S., Meric-Bernstam F., Lucci A. (2009). A prospective study comparing touch imprint cytology, frozen section analysis, and rapid cytokeratin immunostain for intraoperative evaluation of axillary sentinel lymph nodes in breast cancer. *Cancer*.

[65] Stovgaard E. S., Tvedskov T. F., Lænkholm A. V., Balslev E. (2012). Cytokeratin on frozen sections of sentinel node may spare breast cancer patients secondary axillary surgery. *Pathology Research International*.

[66] Cserni G. (2012). Intraoperative analysis of sentinel lymph nodes in breast cancer by one-step nucleic acid amplification. *Journal of Clinical Pathology*.

[67] Shigematsu H., Ozaki S., Yasui D. (2018). Comparison of CK-IHC assay on serial frozen sections, the OSNA assay, and in combination for intraoperative evaluation of SLN metastases in breast cancer. *Breast Cancer*.

